# Brain metastasis with subtype conversion in a patient with male breast cancer: A case report

**DOI:** 10.1097/MD.0000000000024373

**Published:** 2021-03-19

**Authors:** Byeong Ho Oh, Chang Gok Woo, Youn Joo Lee, Young Seok Park

**Affiliations:** a Department of Neuroscience, Graduate School, College of Medicine, Chungbuk National University, Cheongju, Republic of Korea; b Department of Neurosurgery; c Department of Pathology, Chungbuk National University Hospital, Cheongju; d Department of Radiology, Daejeon St. Mary's Hospital, The Catholic University of Korea; e Department of Neurosurgery, Gamma Knife Icon Center, Chungbuk National University Hospital, Cheongju, Republic of Korea.

**Keywords:** Brain metastases, Male breast neoplasm, Pathology, Subtype conversion

## Abstract

**Rationale:**

Brain metastasis of male breast cancer is extremely rare, and the pathological changes between the primary tumor and the metastatic brain tumor have not been reported. Herein, we report for the first time a case of male breast cancer with metastasis to the parietal lobe with subtype conversion after metastasis.

**Patient concerns:**

we describe a 45-year-old male patient admitted for an incidentally found brain tumor after a motorcycle accident. The patient had been treated for breast cancer 5 years previously. The primary tumor was an invasive ductal carcinoma classified as pT1N1M0 with hormone receptor positivity (estrogen receptor ++, progesterone receptor +++, human epidermal growth factor receptor-type2 (HER2) +) and was treated with surgery, adjuvant chemotherapy, radiation therapy and endocrine therapy (tamoxifen).

**Diagnoses:**

Magnetic resonance imaging revealed a well enhanced focal solid tumor in the right parietal lobe (5.0 × 4.2 cm in size), Immunohistochemical staining revealed cerebral metastases of breast cancer with HER2 subtype conversion (estrogen receptor +++, progesterone receptor +++, HER2 −).

**Interventions:**

The patient was successfully treated with surgery and whole brain irradiation (3 Gy × 10 fractions).

**Outcomes:**

There was no additional complication after the surgery and the patient transferred to oncology department for chemotherapy. 2 years later, he had gamma knife radiosurgery due to the recurred brain lesion and after that he discontinued the treatment and opted for hospice care.

**Lessons:**

Male breast cancer with metastasis to the brain is an extremely rare condition. Although a few similar cases have been reported, subtype conversion in similar cases has not been reported. Therefore, we report this case of a male patient with brain metastasis of invasive ductal carcinoma with HER2 status conversion after metastasis.

## 1 Introduction

Breast cancer in men is a rare disease that has infrequently been the focus of research. Its etiology is unclear, but hormone levels may play a role in the development of this disease.^[5]^

Given that male breast cancer accounts for less than 1% of total breast cancer cases and that the incidence of brain metastasis of breast cancer is 10% to 16%, brain metastasis of male breast cancer is very rare.^[22]^

A number of studies have provided evidence about the instability of hormone receptor and human epidermal growth factor receptor-type2 (HER2) status during the progression of the disease, with a particular focus on the relationship between the primary tumor and metastases. Alterations occur most frequently in the hormone receptor status and Ki67 labeling index, and these changes are usually associated with a worse prognosis.^[15]^

A database search yielded 6 case reports^[1,8,17–19,23]^ describing the treatment of male patients with brain metastasis of breast cancer, (Table 1) but subtype conversion during the course of the disease has not been reported. This report presents the case of a male breast cancer patient with invasive ductal carcinoma (IDC) and HER2 status conversion after brain metastasis. In addition, we reviewed similar previously reported cases and include a brief literature review.

**Table 1:** Previously reported cases of brain metastasis of breast cancer in male patients.

Author/Year	Age	Pathological type	Subtype	Treatment	Location
Van Rijswijk/1997^[23]^	53	IDC	ER+	Tamoxifen	Multiple
Nieder C/2003^[18]^	69	IDC	ER+, PR+, HER2+	WBRT	Multiple
Ressl N/2015^[19]^	52	IDC	ER+, PR+, HER2+	WBRT	Multiple
BadKe Gl. /2015^[1]^	59	IDC	ER+, PR+, HER2-	WBRT	Multiple
Namad T/2016^[17]^	84	IDC	ER+, PR+, HER2-	Not described	Temporal lobe
Fuchinoue Y/2018^[8]^	78	IDC	ER+	Surgical removal + WBRT	Temporal lobe
Present case/2020	45	IDC	ER+, PR+, HER2+ → ER+, PR+, HER2−	Surgical removal + WBRT + CTx	Parietal lobe

Abbreviations: CTx = chemotherapy, ER = estrogen receptor, HER2 = human epidermal growth factor receptor-type2, IDC = invasive ductal carcinoma, PR = progesterone receptor, WBRT = whole brain radiation therapy.

## 2 Case report

A 45-year-old man was treated for breast cancer 5 years prior to this report. The tumor was an IDC classified as pT1N1M0 with hormone receptor positivity (estrogen receptor ++, progesterone receptor (PR) +++, HER2 +) and was treated with surgery (modified radical mastectomy of the right breast), adjuvant chemotherapy (adriamycin and cyclophosphamide), radiation therapy (46 Gy) and endocrine therapy (tamoxifen).

Two years previously, whole-body positron emission tomography/computed tomography revealed multiple small nodules in both lungs, but the patient refused additional treatment and was lost to follow-up.

**Figure 1:**
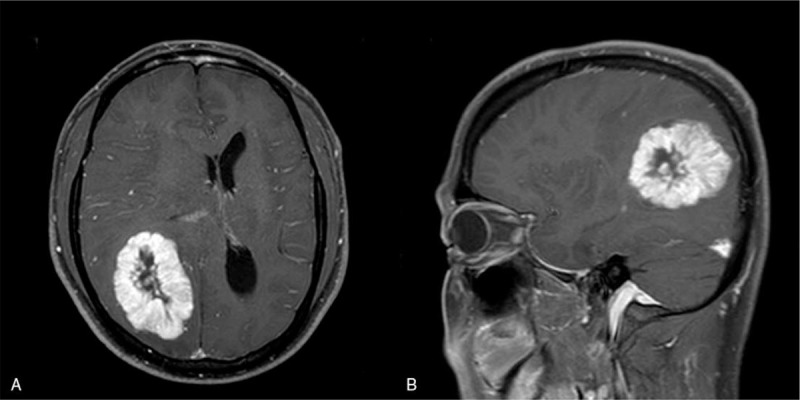
Contrast-enhanced T1-weighted MRI in the axial (A) and coronal (B) planes showed a focal solid tumor in the right parietal lobe (well-defined, round, highly vascular, with surrounding edema, 5.0 × 4.2 cm in size, well-enhanced, with the midline shifted to the left). MRI = magnetic resonance imaging.

**Figure 2:**
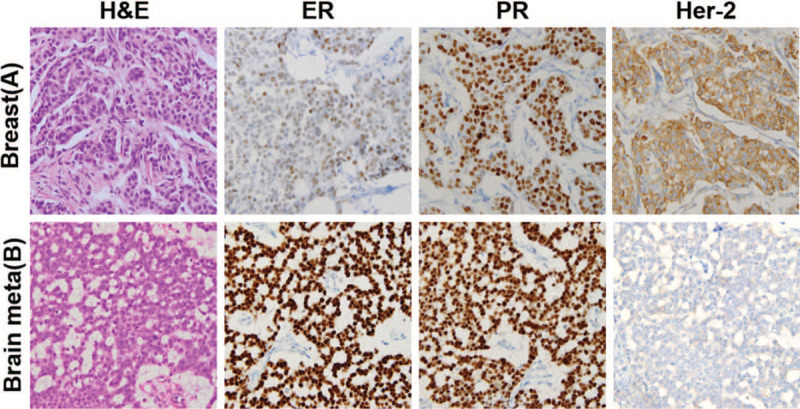
Histopathological and immunohistochemical images from the primary breast tumor (A) and secondary brain metastasis (B) showing HER2 subtype conversion from the primary IDC (ER ++, PR +++, HER2 +) to the brain metastasis (ER +++, PR +++, HER2 −).

Subsequently, the patient presented because of a motorcycle accident, and a neurologic exam revealed right hemianopsia with subtle left side weakness. Magnetic resonance imaging revealed a well-enhanced focal solid tumor in the right parietal area (5.0 × 4.2 cm) with the midline shifted to the left. (Fig. 1) To confirm the pathology and relieve the mass effect, we removed the tumor. Postoperatively, the patient had no additional neurologic deficits. After pathological analysis confirmed brain metastasis of breast cancer, the patient was treated with whole-brain irradiation (3 Gy × 10 fractions for a total dose of 30 Gy), which resulted in palliation of symptoms. Immunohistochemical staining revealed cerebral metastasis of breast cancer with HER2 status conversion (estrogen receptor +++, PR +++, HER2 −). (Fig. 2) After that, the patient transferred to oncology department and received chemotherapy. However, 2 years later, he got gamma knife radiosurgery due to the recurred brain lesion and the chemotherapy regimen was changed due to aggravation of multiple lung metastases. One month later, he discontinued the treatment and opted for hospice care.

## 3 Discussion

Recent review articles have summarized the current knowledge about male breast cancer, a rare disease that is diagnosed mainly in elderly men (median age 63–67 years). Possible risk factors include a positive family history, a low physical activity level and higher-than-average weight and body mass index.^[13]^

Although the definitive etiology is unknown, some diseases that alter the estrogen–testosterone ratio in males predispose to breast cancer; among these diseases, the strongest association is with Klinefelter syndrome. Men with this XXY syndrome have a 50-fold increased risk of breast cancer and account for 3% of all male breast cancer patients. Other diseases and treatments associated with increased estrogen levels, such as cirrhosis and exogenous administration of estrogen (either as hormone therapy in transgender individuals or as therapy for prostate cancer), have been implicated as causative factors. Other risk factors associated with male breast cancer are mutations in the BRCA2 gene and radiation exposure.^[6]^

Most tumors in patients with male breast cancer are IDCs. At the molecular level, there are important gender differences: 95% of male breast cancer patients have luminal A or B tumors, compared with 73% of female breast cancer patients.^[20]^ Both the basal and HER2 phenotypes are rare in men. Regarding genetics, approximately 10% of male breast cancer patients have BRCA2 mutations, and <1% are accompanied by BRCA1 mutations. BRCA2 mutations are found in 4% to 40% of male breast cancer patients compared with 5–10% of female patients with hereditary breast cancer.^[21]^

Prognostic factors include stage, tumor size, lymph node status and histological grade.^[10]^ The survival of male breast cancer patients is comparable to that of matched female patients. Treatment according to the same guidelines used for women is recommended.

Brain metastasis is commonly associated with poor prognosis and diminished quality of life and is normally a catastrophic life-threatening outcome for patients with solid cancers such as breast cancer.^[3]^ Moreover, there are no targeted therapies specific for this secondary tumor formation, and the incidence of brain metastasis is expected to continue to increase.^[2]^

Information about brain metastasis of male breast cancer is insufficient. To the best of our knowledge, only 6 cases of male breast cancer with brain metastasis have been reported. (Table 1).

Breast cancer is a heterogeneous disease, and immunophenotypic changes may occur during progression. As the disease progresses, hormone receptor (especially PR) positivity is usually lost, and mitotic activity increases.^[12]^ HER2 conversion has also been reported in the literature, but it is less frequent than the other phenotypic changes.^[11]^

Despite technical/methodological shortcomings,^[7]^ several biological explanations for subtype conversion have been suggested, for example, tumor heterogeneity or clonal selection of cells with different tumor characteristics.^[9]^ Other reports have shown that contributors to the stromal tissue can perform important breast cancer-related functions that may influence the phenotype, growth, progression and metastatic capacity of the adjacent tumor cells.^[14]^ Furthermore, gene expression studies have reported heterogeneity between the primary tumor and the corresponding metastases (i.e., gene loss, enrichment of mutations and/or new de novo mutations).^[4]^ Additionally, adjuvant therapies may influence clonal selection, resulting in phenotypic differences between metastatic and primary tumor cells.^[16]^

To the best of our knowledge, no research has addressed subtype conversion in male breast cancer. We suggest that there is another mechanism of subtype conversion, because the subtype distribution and BRCA mutation rate of male breast cancer are different from those of female breast cancer.

## 4 Conclusion

Male breast cancer is rare, and brain metastasis of this cancer is extremely rare. Only 6 cases have been reported, but subtype conversion after brain metastasis has not been reported. Therefore, we report this case of a male patient with brain metastasis from IDC with HER2 status conversion after metastasis. In addition, we hypothesize that the mechanism of subtype conversion in male breast cancer is different from that in female breast cancer.

## Author contributions

**Supervision:** Young Seok Park.

**Writing – original draft:** Byeong Ho Oh, Young Seok Park.

**Writing – review & editing:** Chang Gok Woo, Youn Joo Lee, Young Seok Park.
